# Biocontrol of *Sirex noctilio* by the parasitic nematode *Deladenus siricidicola*: A five season field study in southern Chile

**DOI:** 10.1371/journal.pone.0207529

**Published:** 2018-11-15

**Authors:** Miguel Castillo, Eugenio Sanfuentes, Andrés Angulo, Jose Becerra, Jesús L. Romero-Romero, Patricio Arce-Johnson

**Affiliations:** 1 Facultad de Ciencias Forestales, Universidad de Concepción, Concepción, Chile; 2 Forestal Mininco S.A., Los Angeles, Chile; 3 Facultad de Ciencias Naturales y Oceanográficas, Universidad de Concepción, Concepción, Chile; 4 Instituto Politécnico Nacional, CIIDIR, Unidad Sinaloa, Departamento de Biotecnología Agrícola. San Joachín, Guasave, Sinaloa, Mexico; 5 Facultad de Ciencias Biológicas, Departamento de Genética Molecular y Microbiología, Pontificia Universidad Católica de Chile, Santiago, Chile; University of Idaho, UNITED STATES

## Abstract

In 2001, the woodwasp *Sirex noctilio* was detected in *Pinus radiata* plantations in the Biobio region of southern Chile. Subsequently, an intense biological control program using the female sterilizing nematode *Deladenus siricidicola* was implemented in 2010. During five seasons between 2012 and 2017, we studied the parasitism of *D*. *siricidicola* nematode and its effect on woodwasp populations and infestation of *P*. *radiata* in different locations within the Biobio region. Parasitism was assessed by dissecting adult females of *S*. *noctilio* obtained from infested *P*. *radiata* logs. The total population of *S*. *noctilio* was determined by the emergence of individuals from the same logs. The level of damage caused by the *S*. *noctilio* pest was determined by establishing plots in stands of *P*. *radiata* at an intensity of 1 plot every 5 ha-1. During the study period, parasitism of *S*. *noctilio* by the nematode *D*. *siricidicola* increased from 29.6% in 2012 to 93.1% in 2016, while pest population decreased 3.4% in the same time period. Infestation increased from 0.3 to 11,6% of trees between 2012 and 2015, but subsequently decreased to 5.9% by 2017. We confirmed establishment of the nematode in the region under study and its natural dispersion to non-inoculated areas. Finally, we determined that the effect of inoculation age (antiquity) on parasitism levels reached 90% after three years of inoculation.

## Introduction

The wood wasp *S*. *noctilio* Fabricius (Hymenoptera: Siricidae) drills the wood of standing trees belonging to the *Pinus* genus and occasionally *Pseudotsuga*, *Abies*, *Larix* and *Picea* species [1], with the most susceptible species being *Pinus radiata* D. Don [2]. The *Sirex noctilio* attack begins when females oviposit in the stems of live trees and inject a phytotoxic mucus and spores of the symbiotic fungi *Amylostereum areolatum* [1] along with the egg and later the fungus is eaten by the wasp's larva as food. The combined action of the mucus and basidiomycete fungus obstructs the vascular system, resulting in the death of infested trees [3,4]. Moreover, decay associated with the establishment of larvae galleries deteriorates the quality of the wood [5]. The wood-decaying symbiont dries the wood substrate providing a more suitable microenvironment for egg and larvae development. The wood degradation by the fungus facilitates tunneling of the larvae.

*Sirex noctilio* is originally from Eurasia and Northern Africa [3,6] where it presents no ecological or economic damage [7]. However, the pest has a major economic impact in southern hemisphere countries where *S*. *noctilio* has unintentionally been introduced [3], resulting in up to 80% mortality of infested trees in heavily infected areas [8]. *Sirex noctilio* has been reported outside its natural distribution in New Zealand (1900), Australia (1952), Uruguay (1980), Argentina (1985), Brazil (1988), South Africa (1994), Chile (2001) [1,8], the United States (2004), Canada (2005) [9], and China (2013) [10].

In the southern hemisphere, biological control is the most common strategy for management of *S*. *noctilio* [11], particularly with the parasitic nematode *D*. *siricidicola* [8,12]. This nematode, first described by Bedding in New Zealand in 1968 [7], was found to infect *S*. *noctilio* eggs, larvae, pupae, and adults (male and female) [13]. The life cycle of the nematode includes a mycetophagous state in which it feeds on the fungi *A*. *areolatum* and an infective state involving parasitic larvae and the pre-pupae of *S*. *noctilio* [7,8,14,15]. Once inside the host, the female nematode produces juveniles, which develop in the hemocele and migrate to the reproductive organs of *S*. *noctilio* as it pupates. The juveniles subsequently invade the ovaries and eggs, resulting in sterilization of adult female woodwasps and compromising the viability of already laid eggs [16,17]. In the male, the nematode produces a non-sterilizing hypertrophy of the testes [18]. Parasitized *S*. *noctilio* females emerge normally from infested trees and disperse the nematodes [8,19,20].

The first formal biological control program using *D*. *siricidicola* began in the 1970s with work by the Australian Congress of Scientific and Industrial Research Organization (CSIRO), using a nematode strain originally collected in Sopron, Hungary. Subsequently, the Kamona strain replaced the Sopron strain in biocontrol programs [19]. To date, the Kamona nematode has been used for biocontrol purposes in Australia, New Zealand, Brazil, Uruguay, Argentina, Chile, South Africa [3,8] and the United States [19] with variable results [3]. Parasitism levels close to 100% were reported in Victoria, Australia two years after initial nematode inoculation, compared to 90% in Encruzilhado Do sul, Brazil after four years [19] and 96% in Cape Town, South Africa after three years [21]. In contrast, biocontrol programs in Eastern Cape and KwaZulu-Natal, South Africa, reported only 5 to 10% parasitism after two consecutive years of *D*. *siricidicola* inoculation [3]. The varied establishment of *D*. *siricidicola* highlights the need for region-specific control and evaluation programs.

In 2001, *S*. *noctilio* was detected in Chile, and an Official Control Program was developed and implemented in the same year [22]. Currently, *S*. *noctilio* is present in fragmented areas over an approximate surface of 1,400,000 hectares of pine plantations located between the Valparaíso Region (32°30'37.76"S, 71°26'59.42"O) to Aysen 46°49'52.89"S, 71°59'36.12"O [23, 24]. In November 2009, we confirmed the presence of *S*. *noctilio* in *P*. *radiata* plantations belonging to Forestal Mininco S. A. in the Biobio Region. Pest evaluation during 2010 revealed an affected surface of 200 ha, which expanded to 20,000 ha by 2017. Between the months of April and September for the years 2010–2016, *D*. *siricidicola* was successfully inoculated, as has been reported previously [3,8]. Due to the significant economic burden wood wasp represents for the forest industry, coupled with the high cost of biological control of *S*. *noctilio* using *D*. *siricidicola*, we aimed to determine the level of *S*. *noctilio* parasitism by *D*. *siricidicola* over five seasons. In addition, we estimated the effect of parasitism on *S*. *noctilio* populations and resulting levels of infestation in *P*. *radiata* plantations in the Biobio region of Chile.

## Materials and methods

### Inoculation of *Deladenus siricidicola* in *Pinus radiata*

This study was performed between 2012 and 2017 in *S*. *noctilio* infested *P*. *radiata* plantations on the property of Forestal Mininco S.A., located in different sites within the Biobio Region. The extent of *S*. *noctilio* infestation varied annually, with 3,100 ha, 5,800 ha, 11,900 ha, 19,500 ha, 20,000 ha, and 20,000 ha recorded for the years 2012, 2013, 2014, 2015, 2016 and 2017, respectively (unpublished data). Between the months of April and September for the years 2010–2016, *D*. *siricidicola* was inoculated in 2,500, 4,000, 11,455, 12,500, 15,000, 15,000 and 10,000 trees naturally infested by *S*. *noctilio*, respectively. Each tree was inoculated with 250,000 to 300,000 nematodes, as reported previously [3,8]. The inoculation procedure was performed as described by Bedding [8]. Trees were selected for inoculation based on diameter (greater than 15 cm) and the absence of emergence orifices. The presence of *S*. *noctilio* larvae was confirmed through dissection of the stem segment. Trees were debranched and drilled at intervals of 30 cm in two parallel lines along the axe. Cylindrical perforations of 1 cm in diameter were made at a depth of 2 cm. *D*. *siricidicola* was applied in each perforation as a gel suspension. This process was carried out between the months of April to September of each year, without rain and at environmental temperatures ranging between 7°C and 24°C. The temperature or moisture inside the inoculated trees was not measured.

### Selection of sampling sites to evaluate parasitism of *S*. *noctilio* by *D*. *siricidicola*

**Fig 1:**
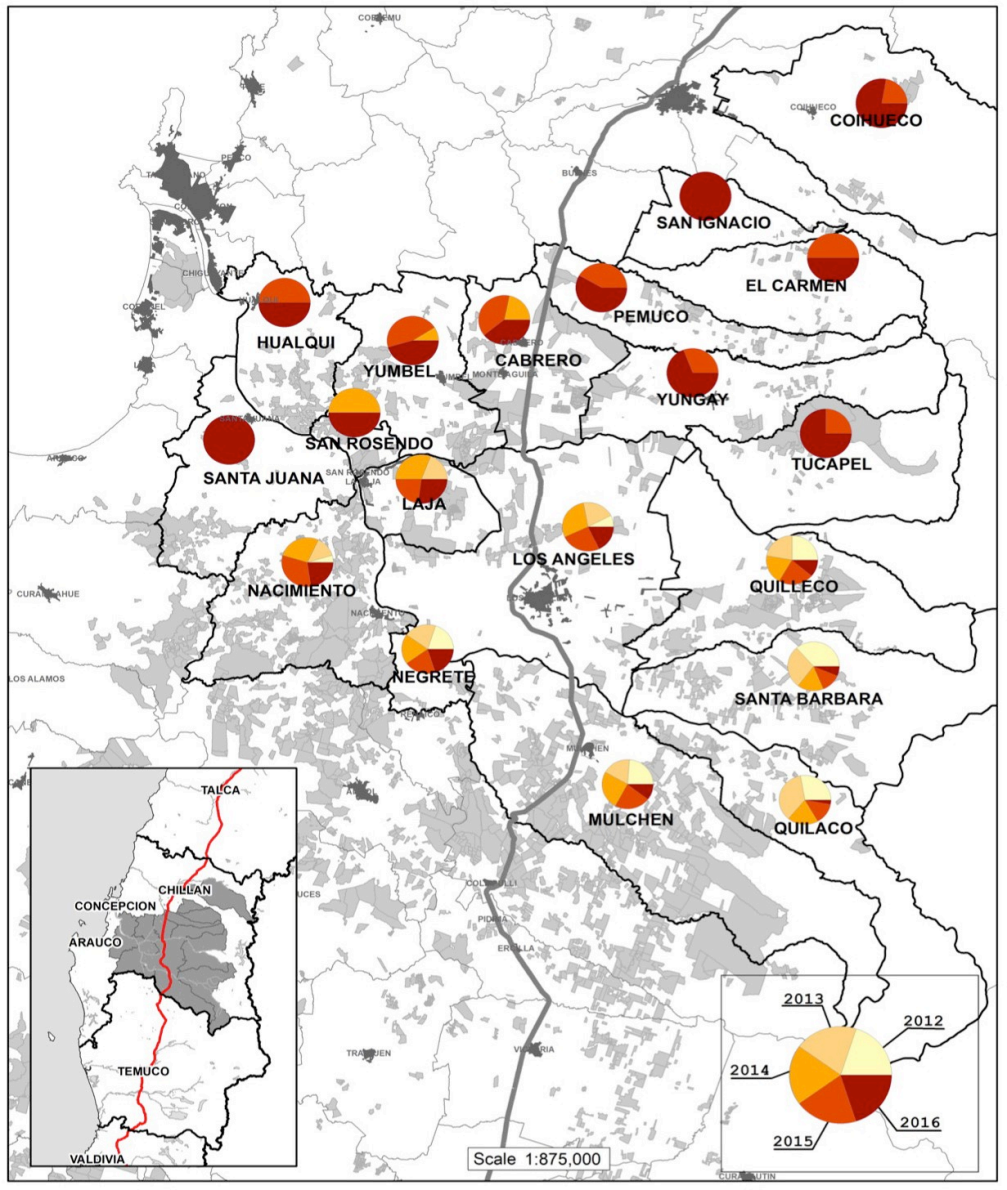
Area of sampling for evaluation of *D*. *siricidicola* parasitism in the Biobío region, Chile. Republished from Luis de Ferrari (personal communication) under a CC BY license, with permission from Luis de Ferrari original copyright 2018. Red line represent the position of Highway.

With the aim of evaluating the efficacy of the biological control program in the macroarea of the Biobio region in southern Chile, sampling were selected across the zone of *S*. *noctilio* colonization, advancing from the south to the north. Fig 1 illustrates a map of the sampling area prepared by Forestal Mininco using the software ArcView 3.2. The first detection of *S*. *noctilio* occurred in 2009 in southern localities with detection continuing through 2015 in northern localities. The Sample Units (SUs) for the evaluation of parasitism of *D*. *siricidicola* were established annually as new localities were colonized by the pest (Table 1) and in stand older than ten years regardless of *D*. *siridicola* inoculation status. Each SU consisted of 3 wooden logs 1 m in length extracted from 3 randomly selected trees with evidence of *S*. *noctilio* infestation. Infestation symptoms include the presence of eggs of *S*. *noctilio* on the tree stem, chlorosis, and fading or death of the foliage [2,5]. Asymptomatic trees were not sampled since they were not counted in the infected population. Therefore, the infestation data represented the population of the pest in infected trees, expressed as individuals attacked/ m^3^. We established 78, 85, 92, 104, and 93 SU for the five years between 2012 and 2016, respectively (Table 1, Fig 1). In some localities, the number of sampling points decreased between years due to reduction of the pest, forest fires, and tree harvests.

**Table 1:** Number of annual sampling units per locality established in *P*. *radiata* plantations to evaluate *D*. *siricidicola* parasitism of *S*. *noctilio*.

Locality	*D*. *siricidicola* *Year of inoculation*		Sampling units (n)			
		2012	2013	2014	2015	2016
Quilaco	2010	10	13	7	5	1
Santa Barbara	2010	35	27	16	12	6
Los Ángeles	2011	6	16	23	20	14
Mulchen	2011	16	12	17	15	7
Nacimiento	2011	1	3	6	7	5
Negrete	2011	1	1	1	1	1
Quilleco	2011	9	8	7	8	4
Laja	2012	.	5	8	6	7
Cabrero	2013	.	.	5	9	9
Coihueco	2013	.	.	.	2	7
Pemuco	2013	.	.	.	5	7
San Rosendo	2013	.	.	1	.	1
Yumbel	2013	.	.	1	5	5
Tucapel	2014	.	.	.	1	3
Yungay	2014	.	.	.	4	9
El Carmen	2015	.	.	.	3	3
Hualqui	2015	.	.	.	1	1
San Ignacio	2016	.	.	.	.	1
Santa Juana	2016	.	.	.	.	3
** **	**Total**	**78**	**85**	**92**	**104**	**93**

Selected trees from each SU were manually felled in October of each year. A meter-long log was extracted from the medial section of the stem. Prior to extraction, the presence of *S*. *noctilio* was determined by splintering the upper and lower ends of the shaft. When the insect was not detected in the stem shaft, the tree was exchanged for another infected individual.

### Evaluation of parasitism of *S*. *noctilio* by *D*. *siricidicola*

*Pinus radiata* logs were labeled and the ends were sealed with solid paraffin until adults hatched according to the methods described by Goycoolea et al. [25]. Logs from the same SU were arranged vertically in a breeding chamber comprised of a cardboard drum (1.1 m of height x 0.7 m width) with a metal mesh cover, avoiding contact between the logs and the walls of the drum. The breeding chambers were kept in a shed with semi-shade mesh (50% coverage) and a polyethylene roof for up to 30 days after the emergence of the last adult *S*. *noctilio* specimen. Breeding chambers were observed weekly and adult *S*. *noctilio* emergence was recorded. *S*. *noctilio* specimens were preserved in 70% alcohol along with to the other individuals that emerged from the same chamber *Deladenus siricidicola* parasitism was determined in the lab by dissecting adult *S*. *noctilio* females using methods described by Zondang [15]. Briefly, the abdomen was cut and placed in a clock glass. Distilled water was added and the abdomen was dissected longitudinally under a dissecting scope (10x). The presence of nematodes was observed directly in the tissue, hemocele, and reproductive organs. Percentage of *S*. *noctilio* parasitism by *D*. *siricidicola* por SU was calculated as a ratio between N° of parasitized in relation to N° of dissected.

### Effect of inoculation antiquity and geography on parasitism of *S*. *noctilio*

To assess the effect of time passed since initial inoculation on parasitism levels, we surveyed parasitism in SU in sites with different inoculation dates. We included SUs from distinct regions (coastal mountain, central valley, and Andes mountain) in consideration of possible geographic differences including Nacimiento from the Nahuebulta mountain chains, Los Ángeles from the South Central Valley, Laja from the North Central Valley and Quilleco from the Pre-Andean site. With the exception of Laja, in which the SUs were inoculated in 2012, all localities were inoculated in 2011. For each SU, we calculated the time in years between inoculation and sampling. For each season, we aggregated parasitism levels for SUs inoculated in the same year. Data was analyzed using the Duncan multiple comparison test (p≤0.05) executed in the R version 3.4.2

### Evaluation of the effect of *D*. *siricidicola* parasitism on *S*. *noctilio* population and *P*. *radiata* infestation

We selected the localities Nacimiento, Los Ángeles, Quilleco, and Mulchén, which were inoculated with *D*. *siricidicola* in 2011, to allow for the longest possible temporal analysis of parasitism evaluation, *S*. *noctilio* population, and *P*. *radiata* damage assessment. *Sirex noctilio* population was determined according to the emergence of adult specimens in the growth chambers and expressed as the number of *S*. *noctilio* specimens per cubic meter of infected trees. The volume was obtained by applying the formula for a cylinder.

*Sirex noctilio* infestation of *P*. *radiata* was evaluated between 2012 and 2017. The infestation evaluation unit was comprised of lineal parcels of 30 trees. Evaluation units were established every five hectares. *Sirex noctilio* infestation was confirmed according to the signs and symptoms described by Newmann et al. (1987) and Aguilar & Lanfranco (1988) [2,5]. Results were expressed as the percentage of infested trees versus healthy trees (i.e. infestation intensity). The relationship between infestation intensity by *S*. *noctilio* and *D*. *siricidicola* parasitism was analyzed using the Duncan multiple comparison test as described above.

## Results

### *Sirex noctilio* emergence

Total adult *S*. *noctilio* emergence (male and female adult specimens) between 2012 and 2016 was 3,374, 2,259, 4,033, 3,100 and 1,597 specimens, respectively. The specimens/m^3^ in the 2012 was of 478.3 (±22.4), in the 2013 was of 374.5 (±25.1), in the 2014 was 721.5 (±41.1), in the 2015 was 678.9 (±38.3) and in the 2016 was 461.7 (±22.9), respectively.

**Fig 2:**
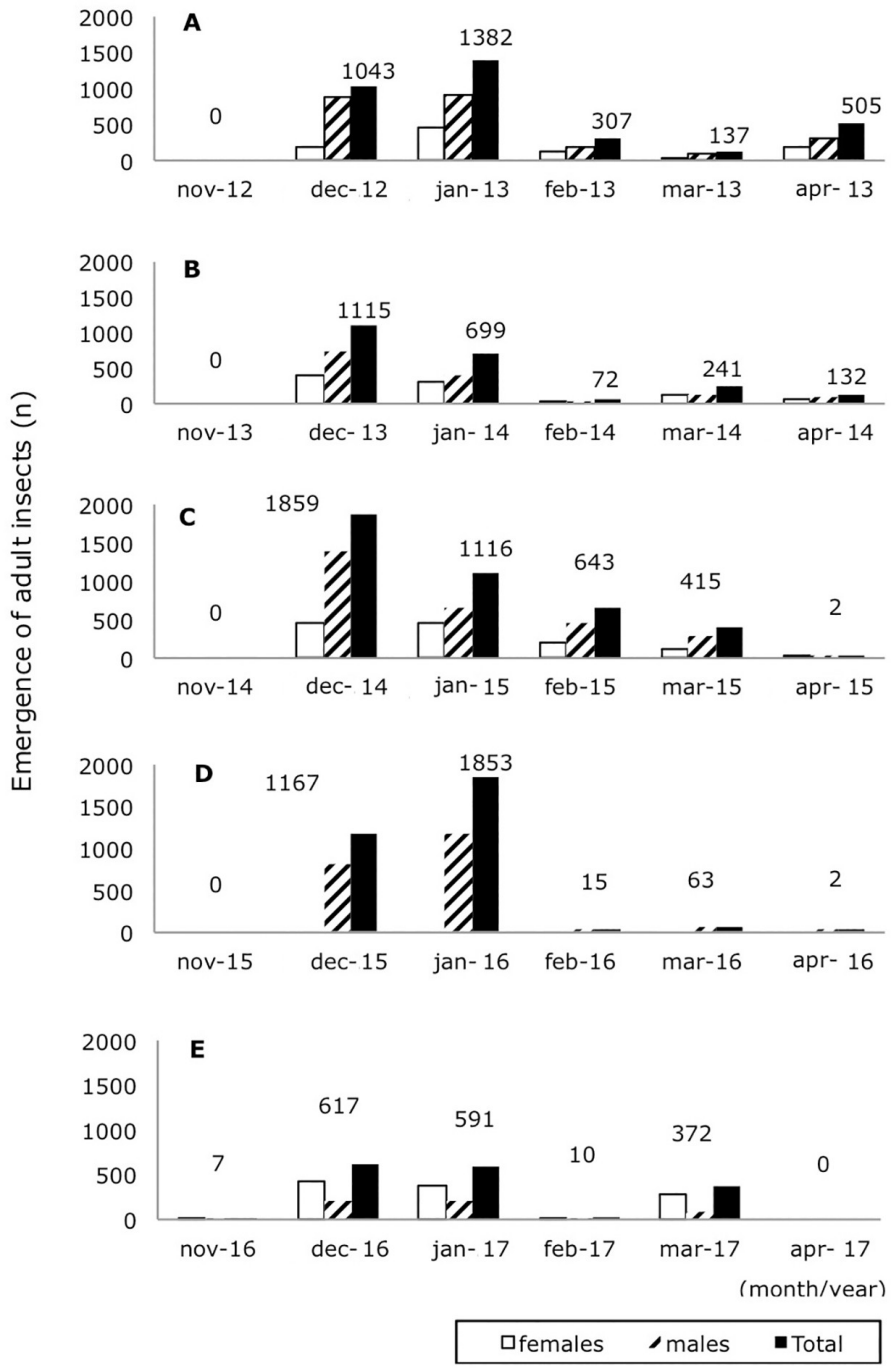
Seasonal emergence of adult *S*. *noctilio*. (A) Season 2012–2013; (B) Season 2013–2014; (C) Season 2014–2015; (D) Season 2015–2016, (E) Season 2016–2017.

During the first four seasons, *S*. *noctilio* adult emergence occurred between December and April. During the fifth season, adults emerged between November and March. For the 2012–2013 and 2015–2016 seasons, emergence peaked in January, with 41% and 60% of total emergence occurring during that month, respectively. For all other seasons, emergence was highest in December. Until the fourth season, male emergence was higher than female emergence with male to female ratios of 2.5:1, 1.6:1, 2.2:1, 2:1, and 1:2 for each season, respectively (Fig 2)

### *Deladenus siricidicola* parasitism of *S*. *noctilio* females

*Deladenus siricidicola* parasitism of *S*. *noctilio* females reached averages of 29.6%, 61.9%, 93.6%, 96.5% and 93.1% for years 2012, 2013, 2014, 2015 and 2016, respectively (Table 2). The number of locations with *D*. *siricidicola* parasitism increased over time, varying from 57% during the first season to 100% in the third season. *D*. *siricidicola* parasitism increased with time in all study locations, exceeding 85% from 2014 onward (Table 2). During 2015, we observed *D*. *siricidicola* parasitism of *S*. *noctilio* in the non-inoculated locations of El Carmen and Hualqui (Table 2). In addition, we observed *D*. *siricidicola* parasitism in several SUs in seven non-inoculated sites in 2012 and nine non-inoculated sites in 2013.

**Table 2:** *Deladenus siricidicola* parasitism of female *S*. *noctilio* by locality during five seasons.

Locality–year of inoculation	Parasitism of *D*. *siricidicola* over female *S*. *noctilio* (%)				
	2012	2013	2014	2015	2016
Quilaco—2010	**53.4** (13.9)^a^	**85.0** (9.1)^ab^	**100.0** (0.0)^a^	**96.7** (3.3)^a^	**100.0** (NE)
Santa Bárbara—2010	**47.6** (7.2)^a^	**89.3** (6.1)^a^	**97.5** (2.5)^a^	**100.0** (0.0)^a^	**100.0** (0.0)^a^
Los Ángeles—2011	**0.0** (0.0)^a^	**31.3** (11.3)^ab^	**90.8** (3.4)^a^	**96.7** (2.9)^a^	**94.4** (0.0)^a^
Mulchén—2011	**17.3** (11.7)^a^	**43.3** (13.7)^ab^	**94.3** (2.7)^a^	**96.9** (2.1)^a^	**100.0** (0.0)^a^
Nacimiento—2011	**0.0** (0.0)^a^	**0.0** (0.0)^b^	**96.9** (3.1)^a^	**90.0** (7.2)^a^	**86.3** (7,9)^b^
Negrete—2011	**0.0** (0.0)^a^	**0.0** (0.0)^b^	**93.3** (0.0)^a^	**100.0** (0.0)^a^	**80.0** (0.0)^a^
Quilleco—2011	**30.8** (12.4)^a^	**53.8** (18.0)^ab^	**95.8** (2.8)^a^	**100.0** (0.0)^a^	**94.7** (5.4)^a^
Laja—2012	**.**	**58.3** (20.6)^ab^	**85.0** (10.6)^a^	**100.0** (0.0)^a^	**85.4** (5.5)^**a**^
Cabrero—2013	**.**	**.**	**100.0** (0.0)^a^	**93.4** (4.2)^a^	**97.9** (1.1)^**a**^
Coihueco—2013	**.**	**.**	**.**	**100.0** (0.0)^a^	**85.7** (14.3)^**a**^
Pemuco—2013	**.**	**.**	**.**	**85.4** (13.8)^a^	**88.9** (4.3)^**a**^
San Rosendo—2013	**.**	**.**	**91.3** (0.0)^a^	**.**	**.**
Yumbel—2013	**.**	**.**	**100.0** (0.0)^a^	**100.0** (0.0)^a^	**100.0** (0.0)^**a**^
Tucapel—2014	**.**	**.**	**.**	**100.0** (0.0)^a^	**97.4** (2.6)^**a**^
Yungay—2014	**.**	**.**	**.**	**98.2** (1.8)^a^	**98.9** (7.7)^**a**^
El Carmen—2015	**.**	**.**	**.**	**100.0** (0.0)^a^	**92.3** (NE)
Hualqui—2015	**.**	**.**	**.**	**91.3** (0.0)^a^	**90.0** (NE)
San Ignacio—2016	**.**	**.**	**.**	**.**	**100.0** (NE)
Santa Juana—2016	**.**	**.**	**.**	**.**	**100,0** (NE)
**Total**	**29,6** (±2,6)	**61.9** (±3,0)	**93.6** (±0,5)	**96,5** (±0,4)	**93,1** (±0,9)

Values in parentheses indicate standard error. NE: Not evaluated

The different letters indicate statistically significant differences between mean parasitism levels per locality within the same year of evaluation (p≤0.05)

### Effect of time since inoculation on *D*. *siricidicola* parasitism of *S*. *noctilio*

Parasitism levels in relation to time since inoculation were different for years 2012 and 2013 in comparison to years 2014, 2015 and 2016. During 2012 and 2013, parasitism levels increased as a function of time since inoculation. For the year 2013, parasitism levels were 52.3, 62.5, and 91.5% for one, two, and three years after *D*. *siricidicola* inoculation, respectively. Table 3 presents data collected from SUs of the 2011 inoculation area. Between the years 2014 and 2016 parasitism levels where higher, more homogenous, and independent of time since inoculation (Table 3). Parasitism levels in relation to time since inoculation increased progressively from the years 2012 to 2014. For one, two and three years after inoculation, parasitism levels increased from 21.5 to 90.9%, 60.7 to 96.9%, and 91.5 to 90.6%, respectively. From 2015 onward, parasitism levels were higher and more homogeneous than previous years with values around 90% (Table 3).

**Table 3:** Female *S*. *noctilio* parasitism according to the age of *D*. *siricidicola* inoculation.

	*Sirex noctilio* female parasitism (%)									

Age of inoculation with *D*.*siricidicola*	2012		2013		2014		2015		2016	
(Years)	(%)	(n)	(%)	(n)	(%)	(n)	(%)	(n)	(%)	(n)
1	**21.5**(6.1)^b^	30	**52.3** (15.2)^b^	11	**90.9** (8.5)^a^	8	**100.0** (0.0)^a^	7	**92.6** (5.5)^a^	18
2	**60.7** (15.2)^a^	5	**62.5** (7.6)^b^	35	**96.9** (3.1)^a^	8	**98.5** (1.5)^a^	7	**94.7** (3.4)^a^	5
3	.	.	**91.5** (7.2)^a^	8	**90.6** (3.7)^a^	28	**100.0** (0.0)^a^	6	**97.9** (1.5)^a^	7
4	.	.	.	.	**100.0** (0.0)^a^	3	**94.3** (3.4)^a^	21	**95.0** (5.0)^a^	5
5	.	.	.	.	.	.	**94.4** (5.6)^a^	3	**82.3** (9.7)^a^	10
6	.	.	.	.	.	.	.	.	**100.0** (NE)	1

Values in parentheses indicate standard error. NE: Not evaluated. The lowercase letter n indicates the number of SUs evaluated. The different letters indicate statistically significant differences between the mean corresponding to time since inoculation (rows) in each year of evaluation (columns) (p≤0.05)

### Geographical distribution on *D*. *siricidicola* parasitism

*Deladenus siricidicola* parasitism of *S*. *noctilio* was determined in four localities representing different geographical zones. No parasitism was observed in Nacimiento from the Nahuebulta mountain chain during the years 2012 and 2013. Los Angeles from the South Central Valley also showed no parasitism during 2012. From the year 2014 onward we observed parasitism in all localities with increased levels of parasitism in comparison to earlier years reaching values ranging between 90.8 and 100% (Table 2). Increases in parasitism levels were generally of greater magnitude between 1 to 2 and 2 to 3 years after inoculation compared to later time points (Table 4). The largest increases were reported in the South Central Valley (59%) during the third year and in the Nahuelbuta mountain chain (90.7%) during the second year (p≤0.05) (Table 4).

**Table 4:** Annual levels of *D*. *siricidicola* parasitism of female *S*. *noctilio*.

Location	Increase in *D*. *siricidicola* parasitism over female *S*. *noctilio* per location (%), between inoculation years.							
	Year (2–1)		Year (3–2)		Year 4–3		Year 5–4	
	(%)	(n)	(%)	(n)	(%)	(n)	(%)	(n)
**North Central Valley**	**22.1** (28.0)^a^	5	**4.4** (4.4)^b^	5	**-10.8** (±6.4)^**b**^	4	**.**	**.**
(Laja)								
**South Central Valley**	**25.0** (19.4)^a^	5	**59,0** (16,0)^ab^	13	**13.5** (5.7)^a^	12	**-2.2** (±1.5)^**ab**^	**10**
(Los Ángeles)								
**Nahuelbuta mountain chain**	**0.0** (NE)^a^	1	**90,7** (9.4)^a^	2	**6.2** (6.2)^a^	3	**3.8** (±6.9)^**a**^	**5**
(Nacimiento)								
**Pre-andean sites**	**45.8** (17.5)^a^	5	**41,3** (19.4)^ab^	5	**8.3** (4.8)^a^	3	**-7.1** (±7.1)^**ab**^	**3**
(Qiilleco)								

Values in parentheses indicate standard error.

The different letters indicate statistically significant differences between the mean from each location within each annual increase (p≤0.05)

### Effect of *D*. *siricidicola* parasitism on *S*. *noctilio* populations and *P*. *radiata* infestation

**Fig 3:**
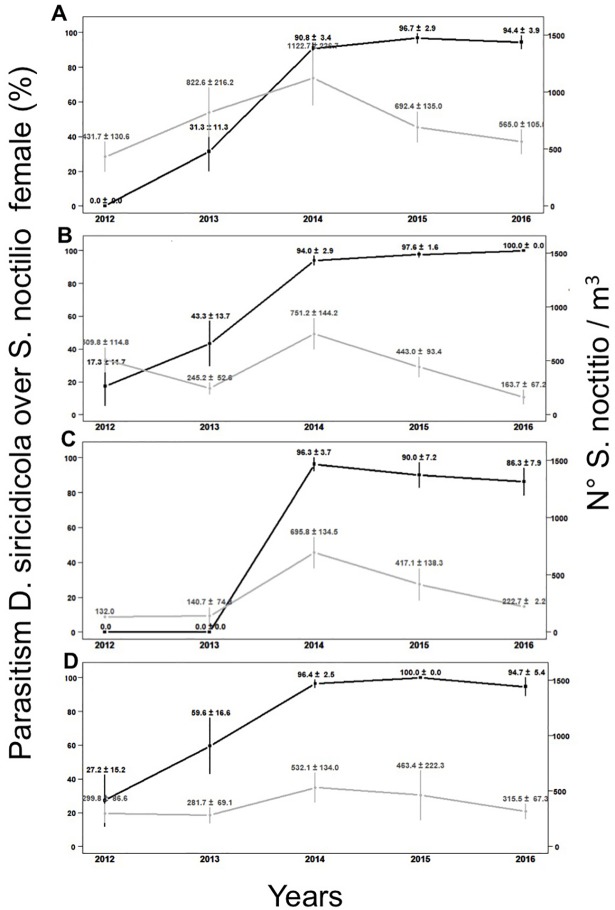
*D*. *siricidicola* parasitism levels and *S*. *noctilio* populations levels between years 2012 to 2016 in A) Los Ángeles, B) Nacimiento,C) Mulchén y D) Quilleco. Black line represent females and grey line represent adults.

The average population of *S*. *noctilio* for the four localities during five seasons between 2012 and 2016 was 408.7, 476.5, 881.5, 544.4, and 394.5 specimens/m^3^ (Table 5). The *S*. *noctilio* population increased from 2012 to 2014. By 2016, the population fell below initial levels, representing an overall 3.4% decrease in the pest density (Table 5). During the third year of assessment (2014), the highest emergence of *S*. *noctilio* was observed during the entire study period. During the same season we observed an increase in *D*. *siricidicola* parasitism levels which then stabilized at 90% in all four localities (Table 2). Between 2015 and 2016, the *S*. *noctilio* population decreased (Fig 3).

**Table 5:** *Sirex noctilio* adult emergence obtained for the SUs from Los Ángeles, Mulchén, Nacimiento, and Quilleco for five seasons.

	*Sirex noctilio*/m^3^ by season				
Locality	2012	2013	2014	2015	2016
Los Ángeles	**431.7**(±130.6)^a^	**822.6**(±216.2)^a^	**1122.7**(±236.7)^a^	**692.4**(±135.0)^a^	**565.0**(±105.5)^a^
Mulchén	**509.8**(±114.8)^a^	**245.3**(±52.6)^b^	**751.2**(±144.2)^a^	**443.0**(±93.4)^a^	**163.7**(±67.2)^b^
Nacimiento	**132.0**(NE)	**140.7**(±74.8)^b^	**695.8**(±134.5)^a^	**417.1**(±138.3)^a^	**222.7**(±22.2)^ab^
Quilleco	**299.8**(±86.6)^a^	**281.7**(±69.1)^b^	**532.1**(±134.0)^a^	**463.4**(±222.3)^a^	**315.5**(±67.3)^ab^
**Average**	**408.7** (21.5)	**476.5** (45.6)	**881.5** (33.3)	**544.4** (17.8)	**394.5** (35.3)

Values in parentheses indicate standard error. NE: Not evaluated. The different letters indicate statistically significant differences between the mean from each location within each season (p≤0.05)

Total *S*. *noctilio* infestation of *P*. *radiata* ranged from 0.3 to 5.9% between the first and last year of assessment (Table 6). For all four localities, *S*. *noctilio* infestation peaked during 2015, coinciding with the season in which the woodwasp population levels started to decrease (Table 5, Fig 3). In each subsequent year, infestation levels decreased (Table 6). *S*. *noctilio* infestation levels started to decrease during 2016, two years after *D*. *siricidicola* parasitism levels stabilized in 90% and one season after of the initial *S*. *noctilio* population decrease (Table 6).

**Table 6:** *Sirex noctilio* infestation of *P*. *radiata* determined in the Los Ángeles, Mulchén, Nacimiento and Quilleco localities during six seasons.

Locality	Sirex associated damage (%)					
	2012	2013	2014	2015	2016	2017
	(%)	(%)	(%)	(%)	(%)	(%)
**Los Ángeles**	**0.0** (±0.0)^a^	**2.9** (±0.6)^a^	**3.7** (±0.4)^a^	**13.5** (±1.5)^a^	**11.6** (±1.4)^a^	**8.9** (±1.9)^a^
**Mulchen**	**0.4** (±0.3)^a^	**3.7** (±1.3)^a^	**2.5** (±0.5)^ab^	**12.6** (±3.3)a	**3.9** (±1.0)^b^	**2.1**(±0.6)^b^
**Nacimiento**		**1.6** (±1.1)^a^	**1.9** (±0.4)^ab^	**9.7** (±1.4)^a^	**8.4** (±1.2)^ab^	**4.3**(±1.2)^ab^
**Quilleco**	**0.4** (±0.4)^a^	**5.8** (±3.4)^a^	**0.7** (±0.4)^b^	**9.3**(±1.1^**)a**^	**8.7** (±3.8)^ab^	**3.1** (±0.9)^b^
**Mean plus SEM**	**0.3 (±0.1)**	**3.2 (±0.2)**	**3.0(±0.3)**	**11.6 (±0.2)**	**9.2 (±0.2)**	**5.9 (±0.4)**

Values in parentheses indicate standard error. The different letters indicate statistically significant differences between the mean from each location within each season (p≤0.05)

## Discussion

Evaluating *D*. *siricidicola* parasitism of *S*. *noctilio* revealed a continuous increase in the frequency of *D*. *siricidicola* detection and parasitism, reaching 100 and 93.6%, respectively, from 2014 onward. *D*. *siricidicola* nematode samples where obtained from naturally infested trees suggesting establishment of the nematode in the area as well as the probable natural dispersion through flight periods of the females as suggested by Taylor [20]. The mean parasitism levels reported during the last three seasons of evaluation (93.6%, 96.5%, and 93.1%) are closer to those reported in South Africa (96% in Cape Locality) and Brazil (90% in Encruzilhada Do Sul) than to the 100% parasitism levels reported for Victoria, Australia two years after inoculation [8]. The parasitism levels observed in this study were also higher than those reported in the US by Williams and Hajek [19], which ranged from 20.5–28.1% and 13.6–17.6%.

The wide fluctuation in parasitism levels between locations during the first two seasons of assessment (0 to 89%) suggests that *D*. *siricidicola* was in the middle of the colonization process. This situation changed in 2014 when parasitism levels were higher (up to 100%) and more homogeneous. The apparent absence of *D*. *siricidicola* during 2012 and 2013 could be due to several factors. Consistent with the density dependence question raised by Bedding [8], the low levels of inoculation after one year coupled with low *Sirex* populations could have rendered *D*. *siricidicola* parasitism undetectable. *Deladenus siricidicola* parasitism was determined under a dissecting scope, a sensitive method to detect the presence of a nematode. Assuming that parasitism existed in the field, the number of samples may have been an insufficient representation of a population with low nematode presence. Moreover, *D*. *siricidicola* inoculum may vary in their parasitic ability as reported by Yu et al [7] in Canada, Williams and Hajek [19] in the US, and Bedding [8] in Australia. However, the viability of inoculum as well as the inoculation technique was similar to those reported in successful cases.

The interaction between nematodes and the symbiotic fungus *A*. *areolatum* within *S*. *noctilio* was not analyzed in the present study. It’s has been reported that nematode reproduction in *D*. *siricidicola* can be negatively affected by a unique mechanism of parasitism of adults and eggs by the fungus *A*. *areolatum* [26]. This discovery provides a possible explanation why *Deladenus* does not survive in culture when *Amylostereum* is fast growing or when the ratio of nematodes to fungus is inordinately biased toward the fungus [26]. This fungus has been shown to interfere with biological control of wood wasp, but in this work, the presence of *A*. *areolatum* was not observed. The insect *Ips grandicollis* has also been reported to effect biocontrol of wood wasps [27] but to our knowledge, this insect has not been reported in Chile.

Overall, the estimated population of *S*. *noctilio* decreased by 3.4% from 2012 to 2016. The population increased and peaked in the third season, then progressively decreased from the fourth season until the fifth, reaching population numbers lower than initial levels in 2012 (Table 5). This is consistent with the results of Williams and Hajek [18] in the US, where the densities of *S*. *noctilio* were 1,000 Sirex/m^3^ and 300 Sirex/m^3^ in 2007 and 2012, respectively.

Infestation increased from 0.3 to 11.6% of trees between 2012 and 2015 followed by decreases in subsequent years reaching as low as 5.9%. The decline in infestation occurred one year after the pest population decreased. The inflection points in the curves of population density and infestation occurred after one season and two seasons, respectively, following the stabilization of *D*. *siricidicola* parasitism levels of *S*. *noctilio* at 90% in each of the four localities.

The flight period was detected between November and April in this study consistent with the range of flight period from October to May reported by Ruiz [28] in the southern hemisphere and with the October to April period indicated by Iede et al. [29] in Brazil. The 2:1 male to female emergence ratio reported during the first two seasons of the study indicated that the wood wasps were in the process of colonization [28] and reached stability during the last season analyzed.

In this study no effect of the zone on the level of parasitism was determined. The similarity in levels of parasitism observed between Nahuelbuta, the Central Valley (north and south), and the Andean Precordillera as of 2014 was unexpected. *Deladenus siricidicola* parasitism was to increase more slowly in the central valley as the product of a larger pest population; however, parasitism in the Central Valley (Los Angeles) was comparable to that of the other zones. This is due to the high parasitic capacity of *D*. *siricidicola* at high densities of the pest [8, 19, 30] Notably, climatic variations between zones were not significant, with average temperatures for the period under study of 14.1°C, 15°C, and 14°C for the Precordillera, Central Valley, and Nahuelbuta, respectively.

Catastrophic levels of damage caused by *S*. *noctilio* were expected in the study area, particularly in the Biobio region. However, economic losses reported to date have not significantly compromised the forest industry in Chile (personal communication). These results are promising in terms of controlling *S*. *noctilio* populations, especially given the observed penetrance of the nematode *D*. *siricidicola*. Nonetheless, long-term monitoring of *S*. *noctilio* must be considered to detect and prevent potential outbreaks. During the last year of the study, the general level of parasitism decreased from 96.5 (± 0.4) in 2015 to 93.1 (± 0.9) in 2016. This observation is critical to understanding the dynamics of the nematode and wood wasp relationship and the long-term biocontrol of the pest. Achieving high levels of parasitism may come at the cost of reducing nematode presence in the environment due to reduction of the wood wasp host and its role as a dispersion medium for the nematode. Given that the success of nematodes as a biocontrol measure is density-dependent [8], drastically reducing the wood wasp population would likely limit the range and propagation of the nematode, which could affect the sustainability and long-term success of the biocontrol program.

## Conclusions

The nematode *D*. *siricidicola*, parasite of the woodwasp *S*. *noctilio*, has been successfully established in the study area of the Biobio region in southern Chile, dispersing naturally into pine plantations where the nematode was not actively inoculated. Parasitism levels of *S*. *noctilio* by the nematode *D*. *siricidicola* increased progressively from inoculation until the third year following inoculation to eventually stabilize around 90% of parasitism. Three years after inoculation, *D*. *siricidicola* was established in similar levels in all three regions including Pre-Andean sites, the Central Valley, and the Nahuelbuta mountain range in the Biobio region. Our work demonstrates the reduction of *S*. *noctilio* populations and associated decrease in *P*. *radiata* infestation after parasitism by the nematode *D*. *siricidicola* reached 90% in all localities and serves as a useful example of the scale and timeframe over which biological control of *S*. *noctilio* is possible.
